# A microfluidic based biosensor for rapid detection of *Salmonella* in food products

**DOI:** 10.1371/journal.pone.0216873

**Published:** 2019-05-14

**Authors:** Jiayu Liu, Ibrahem Jasim, Zhenyu Shen, Lu Zhao, Majed Dweik, Shuping Zhang, Mahmoud Almasri

**Affiliations:** 1 University of Missouri–Columbia, Electrical and Computer Engineering, Columbia, MO, United States of America; 2 University of Missouri–Columbia, School of Veterinary Medicine, Columbia, MO, United States of America; 3 Lincoln University, Department of Life and Physical Sciences, Jefferson City, MO, United States of America; Florida International University, UNITED STATES

## Abstract

An impedance based microfluidic biosensor for simultaneous and rapid detection of *Salmonella* serotypes B and D in ready-to-eat (RTE) Turkey matrix has been presented. Detection of *Salmonella* at a concentration as low as 300 cells/ml with a total detection time of 1 hour has been achieved. The sensor has two sensing regions, with each formed from one interdigitated electrode array (IDE array) consisting of 50 finger pairs. First, *Salmonella* antibody type B and D were prepared and delivered to the sensor to functionalize each sensing region without causing any cross contamination. Then the RTE Turkey samples spiked with *Salmonella* types B and D were introduced into the biosensor via the antigen inlet. The response signal resulted from the binding between *Salmonella* and its specific antibody demonstrated the sensor’s ability to detect a single type of pathogen, and multiple pathogens simultaneously. In addition, the biosensor’s selectivity was tested using non-specific binding of *E*. *coli* O157 and *E*. *coli DH5 Alpha* while the IDE array was coated with the *Salmonella* antibody. The results also showed the sensor is capable to differentiate low concentration of live *Salmonella* cells from high concentration of dead *Salmonella* cells, and high concentration of *E*. *coli* cells. A detailed study on antibody immobilization that includes antibody concentration, antibody coating time (0.5–3 hours) and use of cross-linker has been performed. The study showed that *Salmonella* antibody to *Salmonella* antigen is not a factor of antibody concentration after electrodes were saturated with antibody, while the optimal coating time was found to be 1.5 hours, and the use of cross-linker has improved the signal response by 45–60%.

## Introduction

The Center for Disease Control and Prevention (CDC) estimates that around 48 million people in America get sick, with 128,000 hospitalizations and 3,000 deaths due to foodborne diseases annually [1]. *Salmonella* is ranked first out of the five major pathogens that contribute to domestic foodborne illnesses resulting in hospitalization and death [2]. In 2013, the Economic Research Service (ERS) from USDA has reported that the annual cost due to infection of *Salmonella* in food source is estimably 3.6 billion in US dollar, while the aggregate cost due to food recall is 77 billion for each year [3]. Among all the foodborne pathogens, *Salmonella* typhimurium is the second most common serotype of *Salmonella* found in humans [4]. A method that can provide rapid, selective and accurate detection of *Salmonella* in food products is needed for better food safety.

Currently, microbiological culture and colony counting as a traditional method is still the most widely used technique for the food industry [5]. This method heavily relies on bacteria enrichment and subsequent colony counting [6, 7]. FDA established it as the official food screening procedure for clinical and food production pathogens detection [8]. However, a definitive result usually requires 2–5 days of professional work, which makes this method time consuming, labor intensive and costly [9]. Nucleic acid based assays and their relevant techniques such as PCR, qPCR [10], mPCR [11] are well established for rapid and accurate pathogen detection in food products with high specificity and low detection limit [12]. It is popular in the food industry because it reduces the diagnosis time to 24 hours [13]. However, if the processing plant/company does not have its own lab, additional time is needed to transport samples to a lab that can perform PCR [14]. The current detection time based on PCR is still too long, and they may also fail to differentiate between live and dead bacterial cells without additional chemicals and procedures [15], thus leading to false positive result [16, 17]. Immunological methods such like enzyme-linked immunosorbent assay (ELISA) [18] and their relevant techniques such as IMS-ELISA, ELISA-PCR are based on antibody-antigens binding process. This technique is rapid only after the necessary enrichment culture step, e.g., the commercially available Solus Scientific Testing Solutions can detect *Salmonella* in 36 hours [19]. The long test turnaround time not only cuts into a product’s short shelf-life but also increases the product cost due to the need for storage space and labor needed to transport the products in and out of storage. Alternatively, if food products are released before testing is completed, the company risks releasing product that may cause a foodborne illness or outbreaks of foodborne diseases, economic losses from medical costs associated with foodborne illnesses and product recalls, damage the company’s brand or even survival. Therefore, a testing device/method that could offer a rapid, accurate detection of foodborne pathogens is in urgent need.

Recently, development in biosensors has focused on rapid detection, limit of detection (LOD), feasibility of operation and low cost. These biosensors based on the immunoassay principle can be grouped into three major categories: (1) Electrochemical sensors including: (a) Amperometric biosensor. With present of immunomagnetic beads, a screen–printed carbon electrode has detected *Salmonella* with the LOD of 89 CFU/ml [20]. (b) Potentiometric biosensor. Shaibani *et al* has described a portable nanofiber-light addressable potentiometric sensor for detection of *E*.*coli* with a LOD of 100 CFU/ml within only 1 hour [21]. (c) Impedimetric sensors. A glassy carbon electrode modified with graphene oxide and carbon nanotubes has demonstrated an ability to detect *Salmonella* low to 25 CFU/ml [22]. Wan *et al*, described a signal-off impedimetric immune-biosensor using AuNP to detect *E*.*coli* with LOD of 100 CFU/ml [23]. And Yang *et al*, created label-free impedance spectroscopy biosensor array for alpha-fetoprotein detection with the LOD of 0.1 ng/L [24]. (2) Optical based biosensor which includes: (a) Surface plasmon resonance (SPR). A SPR biosensor based on ultra-low fouling and functionalizable poly brushes has achieved detection of *E*.*coli* and *Salmonella* in hamburger 7.4×10^3^ CFU/ml and 11.7×10^3^ CFU/ml, respectively [25]. 120 CFU/ml of *Staphylococcus aureus* in pure culture was detected by a Localized SPR using gold nanoparticles [26]. (b) Surface enhanced plasmon resonance (SERS). The use of magnetic gold nanoparticles has greatly increased the LOD based on SERS. For example, Detection on *Staphylococcus aureus* and *Salmonella* of 35 CFU/ml and 15 CFU/ml have been demonstrated recently [27]. (c) Colorimetric. For example, Suaifan *et al* has designed an optical colorimetric biosensor for detection of *Staphylococcus aureus* and achieved LOD of 7 CFU/ml in pure culture while 40 CFU/ml in food culture [28]. (3) Mass based biosensor. This includes (a) Acoustic wave-based biosensors. For example, a micro-nano-bio acoustic system using magnetic beads to capture cells has detected *Salmonella* at only 2 cells/μl in 7 hours [29]. (b) Quartz crystal microbalance (QCM) method. For example, a QCM-based platform using ssDNA aptamers has achieved detection of 10^3^ CFU/ml of *Salmonella* within only 1 hour [30]. (c) Cantilever and based biosensor. For example, cantilever biosensor in dynamic-mode without surface functionalization has successfully detected *E*.*coli* at 100 cells/ml [31]. Table 1. shows detection of *Salmonella* using different technologies in various food matrices. Although many of these methods have provided promising research results, in some situations they have relevant drawbacks in terms of detection limit, testing setup, sensitivity and specificity.

**Table 1:** *Salmonella* detection using different technologies.

Detection technique	Sample matrix	Analysis time	Detection limit (CFU/ml)	Ref
Polymerase Chain Reaction	Chicken	24 h	1000	[32]
Enzyme-linked Immunosorbent Assay	Raw chicken	33 h	1000 CFU/25g	[33]
Electrochemical sandwich ELISA	Meat	20h	10 Cells/25g	[34]
Surface Plasmon Resonance	Chicken rinse	3 min	10^6^	[35]
Surface-enhanced Raman spectroscopy	Cucumber	3 h	15	[26]
Electrochemical biosensor	Chicken rinse	15 min	100	[36]
	Meat	1.5 h	1000	[37]
Quartz Crystal Microbalance	Chicken meat	10 min	100	[38]
	Chicken rinse	1 h	100	[39]

Impedimetric based biosensor [40], as one kind of the electrochemical biosensors, has shown its promising advantages for detection of foodborne pathogens in terms of detection speed, accuracy, and sensitivity [41]. Such biosensors will have impedance of the electrodes changed in response to captured bacterial cells on electrodes.

In this study, an impedance-based biosensor for simultaneous detection of multiple *Salmonella* serotypes has been designed, fabricated, characterized, and validated using ready-to-eat turkey (RTE) samples. A detailed study on antibody immobilization which includes antibody concentration, antibody coating time and use of cross-linker has been performed. Detection of *Salmonella Typhimurium* with various concentrations was utilized using both RTE turkey samples and pure culture samples. The sensor’s selectivity using different bacterial cells and capability to differentiate between dead and live cells were also validated. The labeling procedure has been eliminated by immobilization with bioreceptors (in our case antibodies) on the surface of the detection electrode. Impedance change was recorded after selectively capturing the target bacteria by the specific antibody on the surface of the electrodes [42]. An impedance analyzer was used to read the impedance changes.

## Materials and methods

### Biosensor design

**Fig 1:**
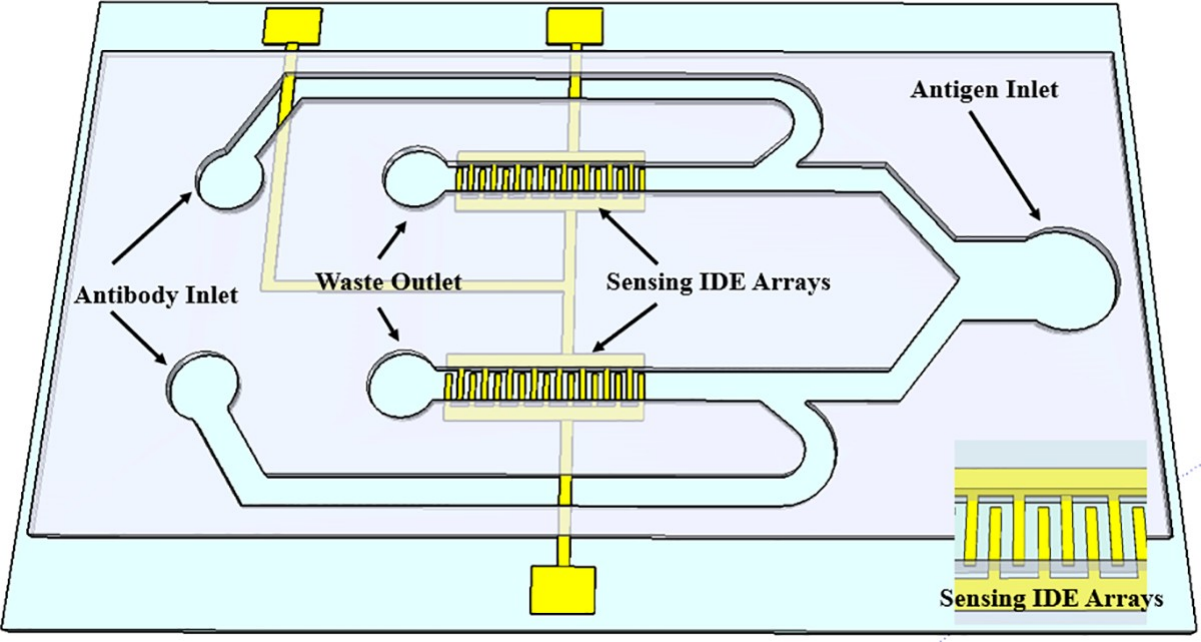
3D schematic of the biosensor showing the two sets of the IDE arrays in two channels. The sensor could be used for sensing two different serotypes of *Salmonella* without causing any cross-contamination. The insertion on the bottom right is the magnified view of the sensing IDE arrays.

**Fig 2:**
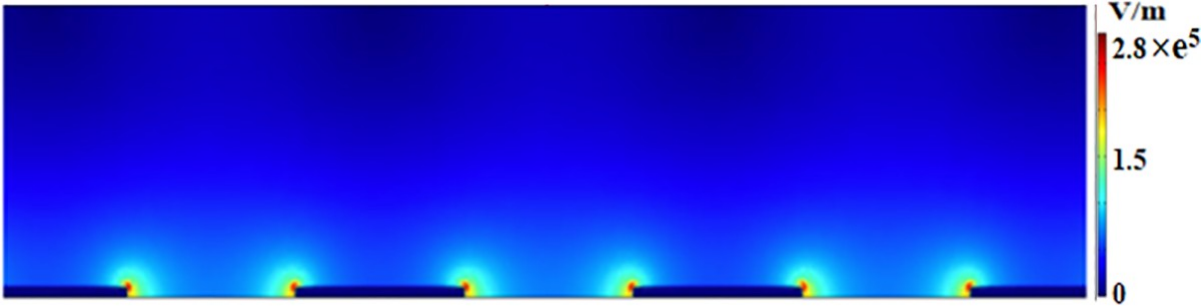
COMSOL simulation on IDE arrays. E-field simulation of the detection interdigitated electrode array at 0.5 mV using COMSOL. The finger width and spacing between fingers are 10 μm and 10 μm, respectively.

The biosensor consists of two detection regions fabricated using SU8 microchannels as follows, Fig 1. Each detection region consists of micro-gaped interdigitated electrode (IDE) arrays fabricated in the same microchannel that incorporates impedance measurement principles to detect the presence/ absence of *Salmonella*. Each electrode arrays consist of 50 finger pairs with finger length, width, and spacing between the fingers are 90 μm, 10 μm, and 10 μm, respectively. A COMSOL simulation were performed in order to determine the optimum dimension of the interdigitated electrode array. The simulation is shown in Fig 2, the modeling results show that the miniaturized dimensions significantly increase the impedance measurement sensitivity, with the spacing between fingers have greater influence on the strength of the E-field intensity compared with the width of the fingers. The bonding pads are stretched away to the sides of the device and used for impedance measurements. The microchannel in the sensing region has a width and height of 100 μm, 25 μm, respectively. The two detection regions were pre-functionalized by flowing a specific anti-*Salmonella* antibody serotypes, B or D, to the electrode through independent antibody inlets without causing cross-contamination. All the antibodies were pre-attached with crosslinker. After the microchannel was filled with antibody solutions, the flow was stopped for 1 hour to ensure efficient adhesion of the antibodies to IDE arrays. The *Salmonella* samples were tested by flowing them through the sample inlet towards the sensing IDE arrays. After the sensing channel was filled with the *Salmonella* samples, the flow was stopped for 30 minutes to facilitate the contact and binding between *Salmonella* antigens and the corresponding *Salmonella* antibodies. It is noted that each device was used for one time only to eliminate the possibility of sample contamination.

### Biosensor fabrication

**Fig 3:**
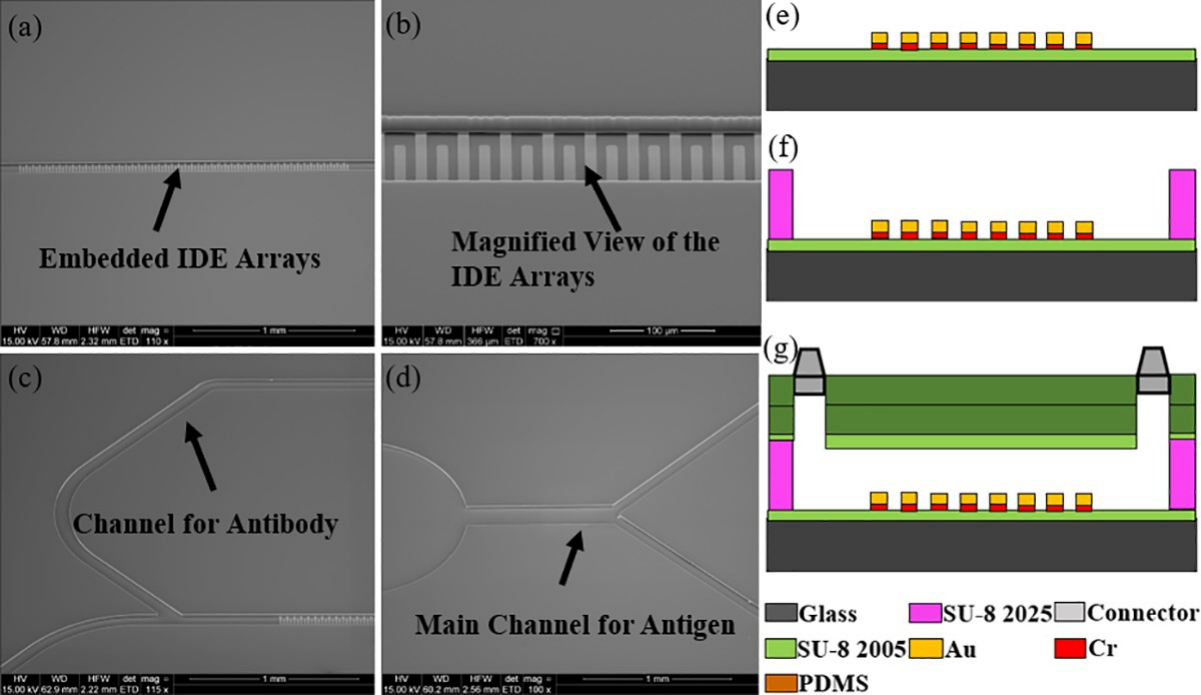
Scanning Electron Micrographs (SEM) of the fabricated biosensor. (a) Embedded IDE arrays in channel. (b) Magnified view of the IDE arrays. (c) The channel for antibody delivery. (d) The main channel for antigen delivery, the channels for sensing region share the main channel during antigen delivery. Cross-sectional view demonstrating multiple layers of the sensor: (e) Electrode traces on top of the SU8 and glass slide. (f) SU8 microchannel. (g) PDMS and fluidic connectors as seal.

**Fig 4:**
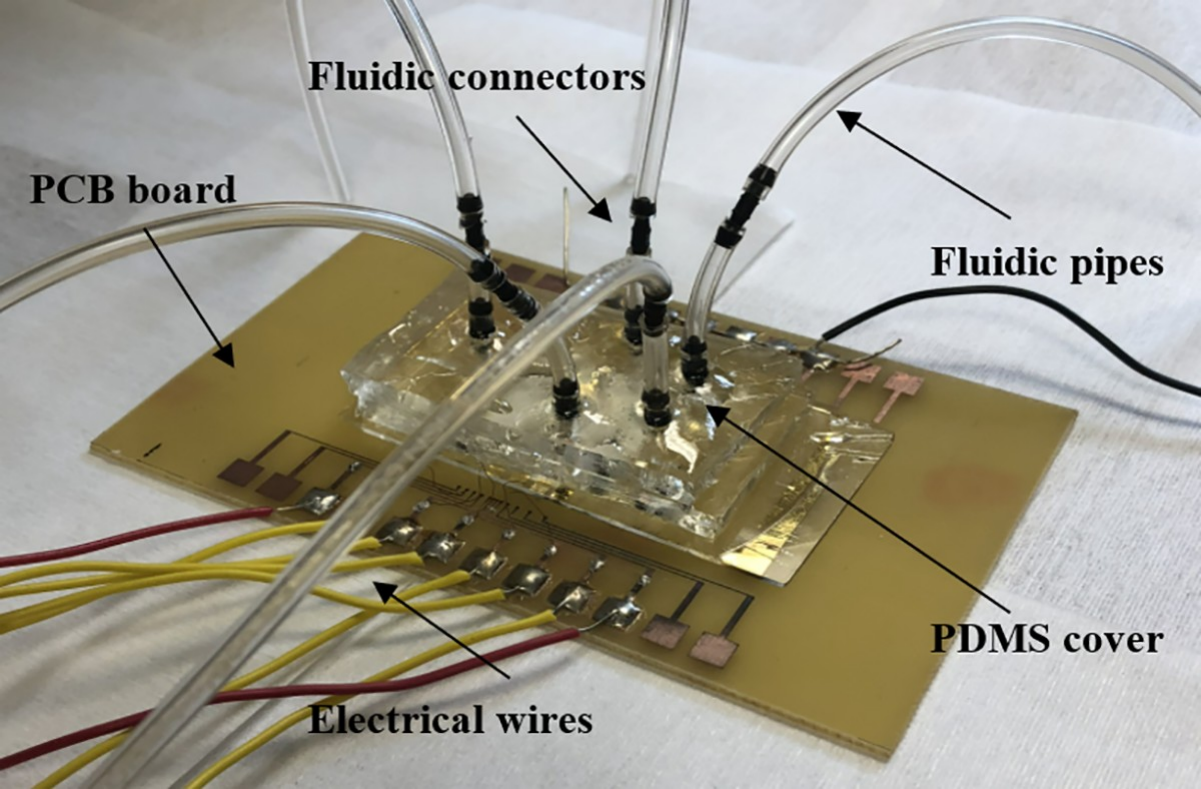
Completely fabricated sensor packaged on a PCB board with the fluidic connectors, tubes and wires.

The biosensor was fabricated on a 2×1.5 inch^2^ glass substrate using surface micromachining in following steps: (1) The glass slide was first cleaned using Piranha solution (hydrogen peroxide and sulfuric acid with a ratio of 1:3) for 4 minutes. (2) A 4 μm layer of SU-8 2005 (Microchem) was spin-coated on the surface of the glass slide following by pre-bake, UV light exposure and hard bake at 150°C for 30 minutes. (3) Chromium (Cr) and gold (Au) thin films were deposited using DC sputtering with the thickness of 50 nm and 150 nm respectively. (4) The Au thin film was patterned using Au etchant to form the IDE arrays and bonding pads. (5) The microchannel was formed using SU-8 2025 negative photoresist with a thickness of 25 μm. (6) Two thick layers of polydimethylsiloxane (PDMS) were prepared and cured at room temperature. The first one is used to cover the microchannel, while the second layer serves as top cover with fluidic connectors. The first layer was treated with oxygen plasma to change its surface to be more hydrophilic before it was aligned and bonded to the glass substrate. Then the bonded PDMS layer and glass slide were baked on a hotplate at 65°C for 5 minutes to ensure the bonding between the PDMS and the glass substrate is secured. The second PDMS slab with inlet and outlet fluidic connectors were then bonded to the first layer of PDMS using oxygen plasma. Epoxy glue was used to seal the fluidic connectors further to improve the device reliability. Scanning electron micrographs (SEMs) of the fabricated devices are shown in Fig 3(A)–Fig 3(D). Fig 3(E)–Fig 3(G) is the fabrication of the sensor step by step. A completely packaged sensor in PCB board in Fig 4 has shown the fluidic connectors, tubes and electrical wires.

### *Salmonella* typhimurium and culture preparation

Ready-to-eat turkey breast (RTE) turkey breast was purchased from a grocery store and stored at 4°C until use. Each 325 g RTE turkey breast was weighted and placed in a sterile bag, 2925 ml buffered peptone water was poured into the bag, and the bag was shaken for one minute. The supernatant was then filtered through a 100 μm and then a 20 μm cell strainer (pluriSelect Life Science, Leipzig, Germany) to remove big debris which may block the biosensor microchannels. The filtered rinse was used freshly to dilute *Salmonella* cells or aliquoted and frozen at -20°C until used. The effect from solution has been minimized because all biological samples were prepared with buffered peptone water.

An avirulent *Salmonella* enterica Typhimurium strain (ΔsipB, Cmr) was then used to spike RTE turkey breast or pure culture. An overnight culture (37°C, 200 rpm, in Luria-Bertani broth) of S. enterica Typhimurium was harvested by centrifugation at 4000 rpm 10 minutes and washing with sterile distilled water 3 times and then suspended in 25% sterile glycerol. The cell suspension was aliquoted and frozen at -80°C until used. At the same time, one aliquot was serially diluted and plated on Luria-Bertani agar plates to determine the cell concentration. The cell concentration was determined to be 2 × 10^9^ CFU/ml. Before the test, one aliquot *Salmonella* suspension and several aliquots of RTE turkey rinse were thawed on ice. The *Salmonella* suspension was then diluted with the filtered RTE turkey rinse to desired concentrations. It is noted that spiked turkey samples do not mimic infections. The focus of the present study is food-safety testing, not disease diagnosis.

Our protocol did not involve grinding meat samples. Filtration through a cell strainer is actually very simple method to remove debris, which only takes less than 15 minutes. Enrichment culture was evaluated because the current food safety guideline requires considerably a large amount/volume of sample (325 g RTE-turkey sample in 2925 ml buffered peptone water). We are comparing our device with the current popular methods. Compared with PCR, our method does not need the DNA extraction and PCR amplification which can be a difficult to animal farms or food processing plants. Compared with culturing method, our method can save bacterial growing time and the following bacterial identification time which are around 1–2 days. The sample preparing (I mean sampling from food) for all methods including our method are all the same. There is a stomaching step involved with the same purpose of grinding. But, the step is only several minutes. For other methods, whether or not having an enrichment, it really depends on bacterial concentrations in the contaminated food. For real application, a test can be conducted before enrichment and a heavy contamination, though it is rare, can be identified in this step. Another test can be conducted after an enrichment to identify a light contamination.

### Antibody preparation

Rabbit anti-*Salmonella* O antiserum poly B, D, and E (Becton, Dickinson and Company, Franklin Lakes, NJ) were used as *Salmonella* antibodies. The crosslinker, sulfosuccinimidyl 6-[3-(2-pyridyldithio) propionamido] hexanoate (sulfo-LC-SPDP) (Fisher Scientific, Hampton, NH), was used for antibody immobilization. Briefly, for each test, 16 μl of each antiserum was diluted with 288 μl filtered chicken rinse, mixed with 300 μl sulfo-SPDP (20 mM water solution), and then incubated at room temperature for 1 hour. To reduce the disulfide bond of the thiolated antibody, 200 μl DTT (0.1 M sodium acetate buffer, 0.1 M NaCl, pH 4.5) (Fisher Scientific, Hampton, NH) was then added into the tube to react for 30 minutes at room temperature before the antiserum mixture was loaded into the biosensor. The antiserum-crosslinker mixtures were then diluted with distilled water in the ratio of 1:100 or 1:50 to form 1× antibody concentration and 2× antibody concentration, respectively.

### Antibody crosslinker

A new immobilization method using cross-linker was used to exploit natural and direct covalent bond formation between gold electrode and a thiolated antibody. The cross-linker we used is Sulfo-LC-SPDP, it is a thiolation reagent that introduce available sulfhydryl groups to an antibody. A monolayer coating of the thiolated antibody onto the gold surface can be easily achieved because the reaction potential between the gold and sulfur is very strong, which achieved 50% in signal response [43, 44]. In our experiments, all the antibodies were prepared with crosslinker.

### Antibody immobilization

**Fig 5:**
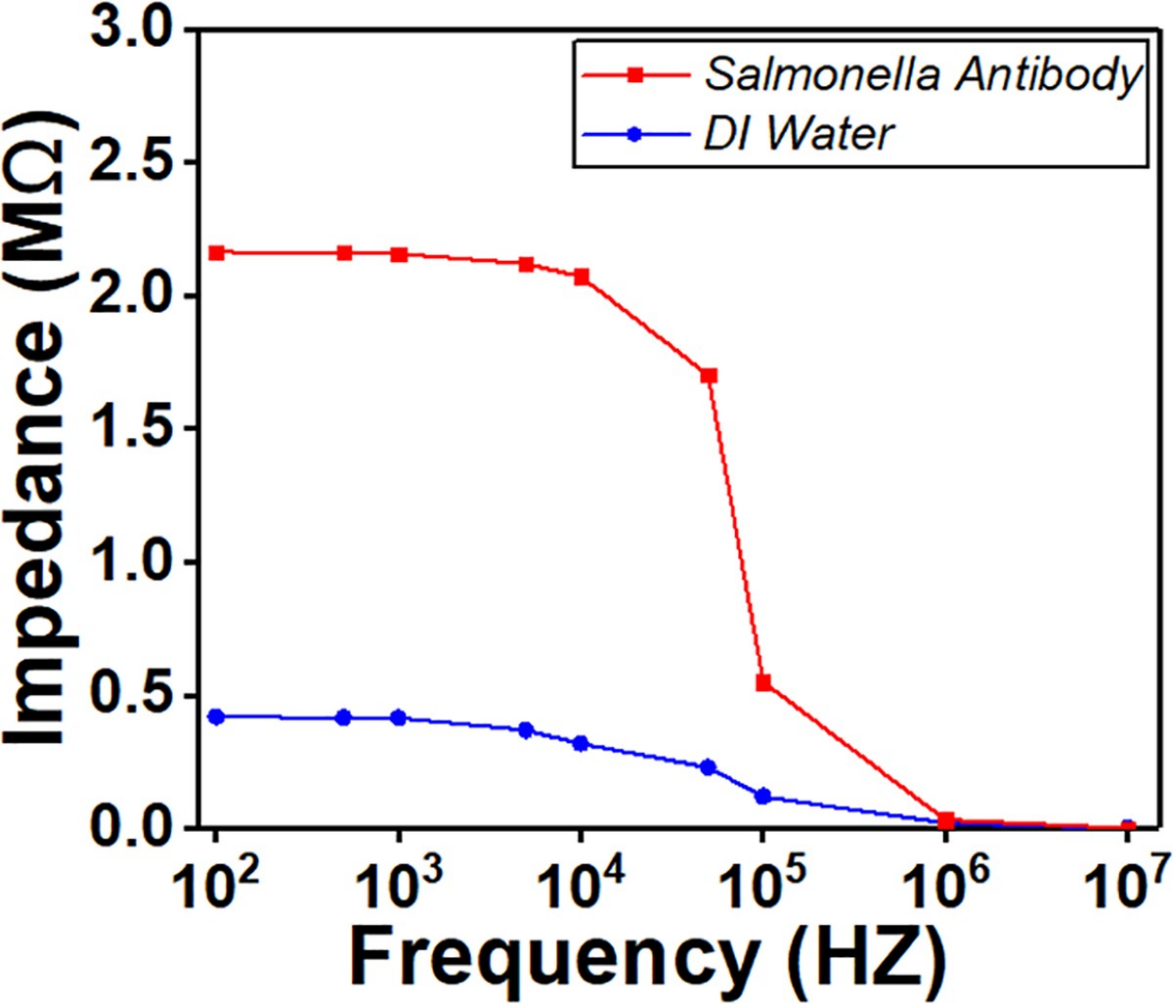
Impedance reading before and after antibody coating. *Salmonella* antibody was prepared with DI water.

The diluted mixtures of antibody with cross-linker were delivered to the sensor through inlets using syringe pumps at a constant flow rate for 15 minutes. Subsequently, the sensing region was then functionalized with two types of antibodies, with one for each sensing region without causing any cross contamination. The flow was stopped for 1.5 hours to allow the antibodies to get firmly immobilized on the gold IDE arrays to achieve a good and specific binding between the antibodies and the gold IDE arrays. A washing step by pumping DI water into the channel was then performed for 30 minutes to remove excess antibodies and other waste materials. Impedance was then measured by an impedance analyzer. The flow rate for all fluids was set to be 2 μL/min to prevent fluids from flowing in random fashion. To confirm antibody adhesion to the IDE arrays, we have measured the impedance of the electrode before antibody coating, and after antibody coating. The impedance change was significant (See Fig 5). The antibody used in all experiment was prepared with DI water. The measured impedance value before antibody coating was less than 0.50 MΩ, while after antibody coating, the impedance value was increased to a value above 2.1MΩ.

### Antigen capturing

The prepared *Salmonella* sample were then introduced into the channel through the sample inlet by a syringe pump at a constant flow rate. After the channel was filled with sample, the flow was stopped for 30 minutes to allow the binding between the *Salmonella* antigens (type B) and *Salmonella* antibodies (type B). Then, the channel was washed again for 30 minutes to remove the unbound or weakly bound antigens and other unwanted particles. The impedance was then measured. The impedance change was calculated by subtracting the measured impedance after antibody coating from the measured impedance after antigen binded with antibody. The impedance difference represents the impedance value of antigens alone. All samples were filtered to remove large food particulates before they were delivered into the sensor. It is noted that the chips are one-time use for all the experiments. The chips are suitable for repeating use with appropriate cleaning process. But the food industry prefers the sensor to be disposable to eliminate the possibility of cross-contamination. In the future when the sensor is manufactured in industry, antibody coating would be a part of the sensor manufacture process. Based on the request from the food industry, the reusability of the sensor was not included in this paper.

#### Data acquisition

Agilent 4294A impedance analyzer was used to record the impedance. A sine wave of 500mV peak voltage was applied across the terminals of the IDM arrays and the corresponding impedance values were measured for frequencies between 100 Hz and 10MHz.

## Results and discussions

### Antibody immobilization study

**Fig 6:**
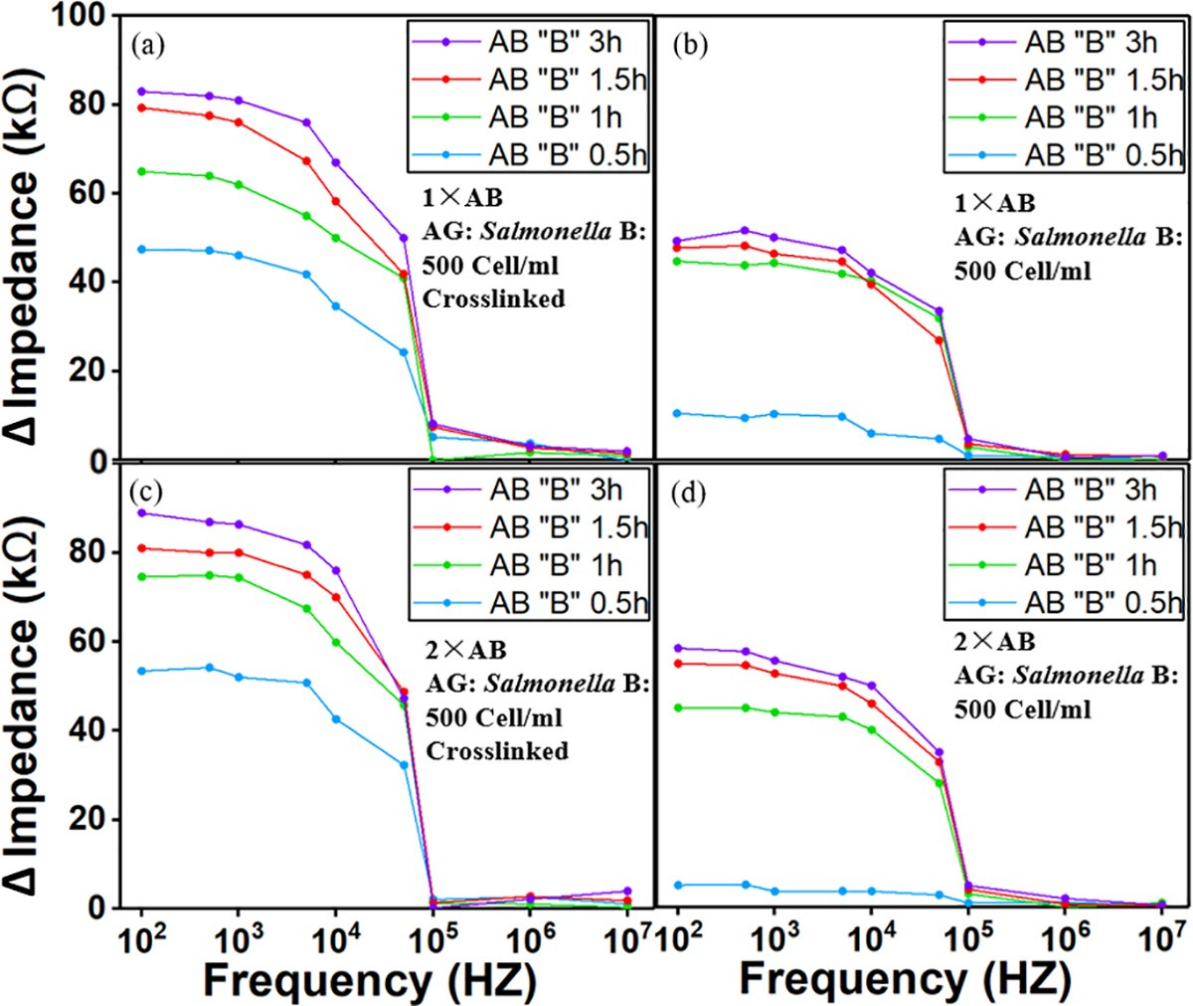
Antibody immobilization study using *Salmonella* in pure culture of 500 Cells/ml. Four different coating time were chosen for study– 0.5, 1, 1.5, 3 hours while the concentrations of antibody are at regular (1×) and double (2×). 1.5 hours was found to be the best time for achieving an efficient and solid binding. There is no significant difference between 1× and 2× antibody concentration on signal response due to saturation of bonding sites on IDE arrays. The use of Sulfo-LC-SPDP as cross-linker has improved the signal response by 50%. AB stands for antibody.

**Fig 7:**
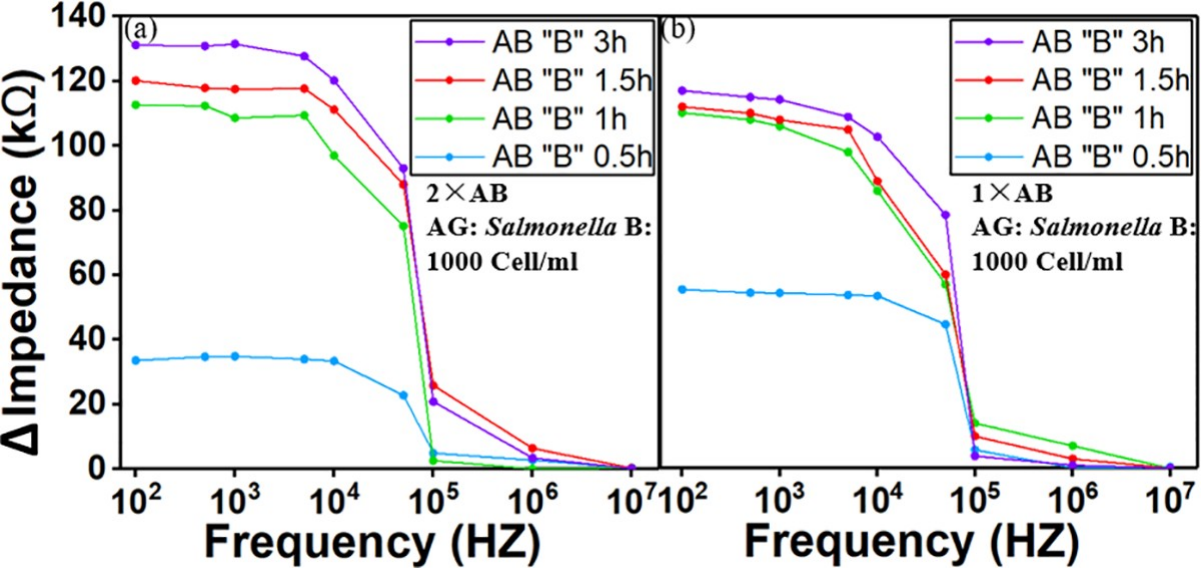
Antibody immobilization study using *Salmonella* in pure culture of 1000 Cells/ml. The results using 1000 Cells/ml are similar to experiments with 500 Cells/ml. In this study, crosslinker was not used.

There are potentially three factors during antibody immobilization that would affect the overall performance of the biosensor: antibody coating time, antibody concentration and use of cross-linker. Fig 6 and Fig 7 show the study on antibody immobilization based on pure culture spiked *Salmonella* of 500 and 1000 Cells/ml. The antibodies were injected from the antibody inlets. Once the detection channels were filled, the flow was stopped for 0.5, 1, 1.5 and 3 hours before the channel/ electrodes are washed to remove the access components. Four different immobilization time ranged from 0.5 to 3 hours were chosen for the study while the antibody concentrations are at single (1X) and double (2X) concentration. It was observed that at 0.5 hours, there is a small change in impedance, while 1-hour coating time seems to provide a higher impedance value which indicates better results from antibody immobilization. The results from 1.5 and 3 hours gave a similar impedance change but higher than the result from 1-hour, which means the binding of antibody to IDE arrays have reached equilibrium. Therefore, 1.5 hours was demonstrated as the best immobilization time for achieving an optimum coating. To study the antibody concentration effect, we tested the biosensor with single and doubled antibody concentration, it was found that the concentration of the antibody doesn’t have a significant effect on impedance response due to saturation of binding sites on IDE arrays to allow absorption of antibodies. For all the experiments, the use of crosslinker has improved the signal response by 50%. It is necessary to have a stable and firm binding of the antibody to electrode for a better sensor stability.

### Dose response with different concentrations of *Salmonella* serotype B

**Fig 8:**
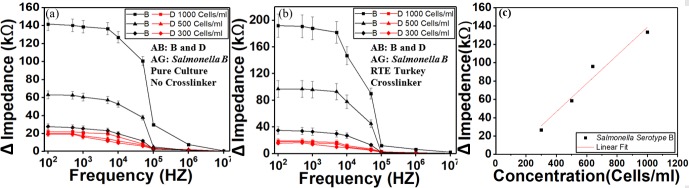
Impedance response for different concentrations of *Salmonella*. (a) Pure culture sample without crosslinker and (b) Ready-to-eat turkey culture with crosslinker. The sensor was first functionalized with *Salmonella* antibody type B and D with crosslinker. Baseline impedance after antibody coating was calculated. *Salmonella* serotype B with concentrations of 1000, 500 and 300 Cells/ml spiked with RTE turkey sample was then delivered to the sensor for detection. Then impedance after antibody was subtracted from the impedance measured after we delivered the antigen to get the impedance change. The limit of detection was found to be 300 Cells/ml. (c) Impedance change as a function of *Salmonella* concentration at 1 kHz. Impedance change is directly proportional (linear) to *Salmonella* concentrations. The linear correlation coefficient is 0.965 and the sensitivity (Slope) is 0.144 kΩ per one *Salmonella* cell. AB stands for antibody. AG stands for antigen.

The variations of the amount of substance on the surface of the electrodes cause the impedance to change. Impedance increases after the antibody is immobilized on the IDE arrays compared to the IDE arrays immersed with just the culture broth. After the *Salmonella* antigens were selectively bound to the *Salmonella* antibody, the impedance was changed again. The variation in impedance response is based on the concentration of the *Salmonella* as shown in Fig 8(A) using pure culture without crosslinker and Fig 8(B) RTE turkey culture with crosslinker. The sensor was first immobilized with *Salmonella* antibody serotype B and D through independent fluidic inlets without causing cross-contamination. Different concentrations of *Salmonella* serotype B was then delivered to the sensor. The results demonstrated a high impedance difference of the electrode with *Salmonella* antibody serotype B while the other electrode where antibody D was coated had a weak signal. The detection limit of the sensor was found to be 300 Cells/ml. For all concentrations, impedance decreases as a function of frequency. Fig 8(C) shows the Impedance change as a function of *Salmonella* concentration at 1 kHz. We found the variation of impedance change is directly proportional (linear) to *Salmonella* concentrations. The linear correlation coefficient is 0.965 and the sensitivity (Slope) is 0.144 kΩ per one *Salmonella* cell. All the sample concentrations were pre-confirmed by the culture method, so the number of bacterial cell colonies is known before being delivered to the sensor for testing. The device cannot quantify the number of detected bacterial cells, but it informs the presence or absence of bacterial cells.

### Simultaneous detection of two types of bacterial cells

**Fig 9:**
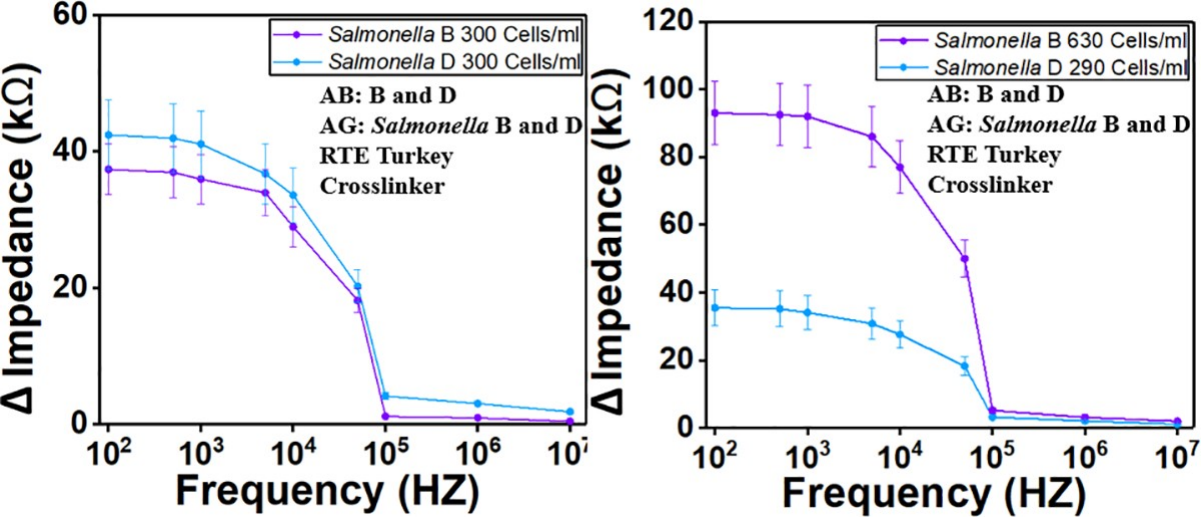
Signal response from mixture of *Salmonella* serotype B and D with two concentration combinations. (a) 600 Cells/ml, 300 Cells/ml and (b) 300 Cells/ml, 300 Cells/ml. AB stands for antibody and AG stands for antigen. Impedance after antibody coating was recorded and subtracted from the impedance after we delivered the antigen to get the impedance change.

In the first set of experiments (Fig 9), two detection regions were coated with the antibody for *Salmonella* serotype B and *Salmonella* serotype D, respectively without causing cross-contamination. Then mixture of *Salmonella* serotype B (300 Cells/ml) and *Salmonella* serotype D (300 Cells/ml) was delivered to the sensor (Fig 9A). A mixture of *Salmonella* serotype B (630 Cells/ml) and *Salmonella* serotype D (290 Cells/ml) was delivered to the sensor (Fig 9B). The results demonstrate that the impedance value was doubled when the *Salmonella B* concentration was doubled, which demonstrated that the sensor can selectively and simultaneously detect multiple pathogens.

**Fig 10:**
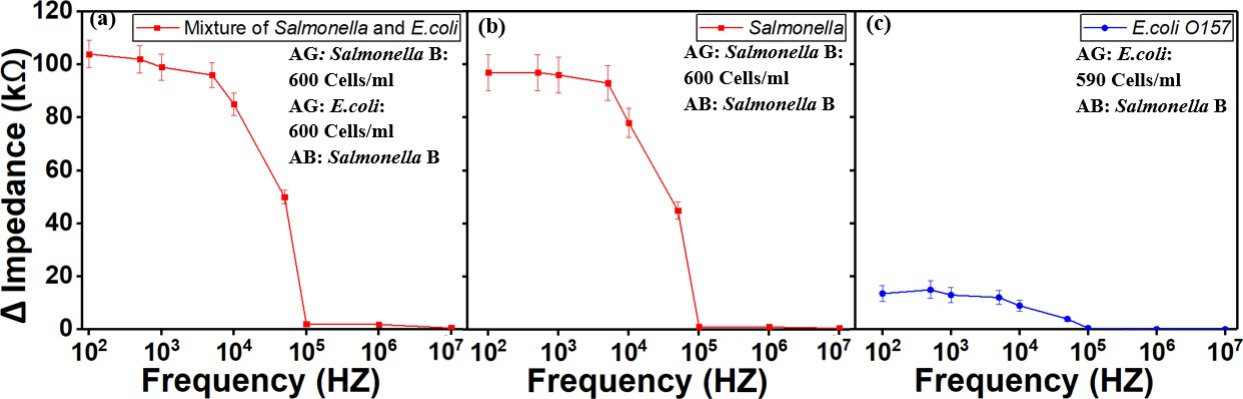
Signal response from mixture of *Salmonella* and *E*.*coli*. All ready-to-eat turkey samples were spiked with pathogens as follow. Crosslinker was used. (a) *Salmonella* mixed *E*.*coli* of 600 Cells/ml was delivered to the sensor when only *Salmonella* antibody was precoated on the detection electrodes. (b) Only *Salmonella* sample was delivered to the sensor when the antibody is for *Salmonella*. (c) Only *E*.*coli* was delivered to the sensor when the antibody is for *Salmonella*. AB stands for antibody. AG stands for antigen.

In the second set of experiments, (a) the two detection regions were coated with antibody for *Salmonella* serotype B, then a mixture of *Salmonella* serotype B (600 Cells/ml) and *E*.*coli O157* (600 Cells/ml) was delivered to the sensor as shown in Fig 10A. (b) Only *Salmonella* serotype B (600 Cells/ml) was delivered to the sensor as shown in Fig 10B. The impedance change values in experiments from (a) and (b) were similar. The mixture of *Salmonella* with *E*. *coli O157* did not increase the impedance value. This indicates that *E*. *coli O157* contamination did not have any effect on the detection of *Salmonella*, and proves the detection is selective. (c) Only *E*.*coli O157* (590 Cells/ml) was delivered to the sensor, the impedance change is low (Fig 10C). The results demonstrated the sensor’s capability to selectively detect target bacterial cell based on the specific type of antibody coating. The nontarget bacterial cell (*E*. *coli O157*) would not bind to the antibodies on electrodes thus would be washed away during the following washing step. The results demonstrated the sensor’s capability to selectively detect target bacterial cell based on the use of antibody.

### Sensor’s Selectivity Using *Salmonella* and *E*. *coli* O157

**Fig 11:**
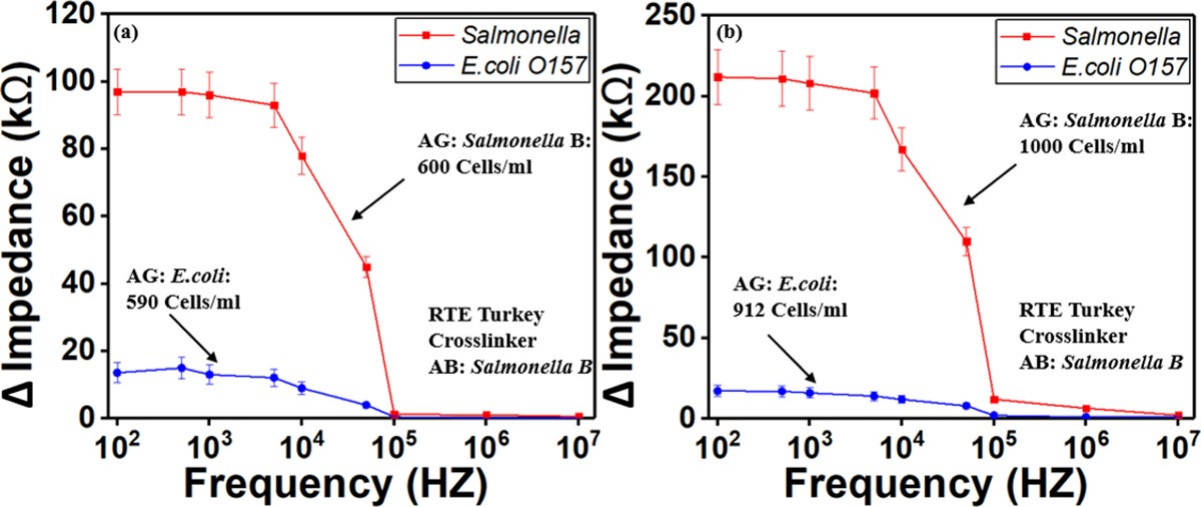
Test of sensor’s specificity to *Salmonella* serotype B for two concentrations. (a) concentration of Salmonella and E.coli around 600 Cells/ml and (b) around 1000 Cells/ml. The result show that E.coli O157:H7 wound not bind to Salmonella antibody due to specificity. RTE turkey spiked sample and crosslinker were used for these experiments. AB stands for antibody. AG stands for antigen.

*Salmonella* and *E*.*coli O157* were used to test the sensor’s selectivity (Fig 11). In the first set experiments, the detection regions were coated with *Salmonella* antibody type B. The RTE turkey samples were spiked with only *Salmonella* type B at different concentrations were introduced into the sensors individually without mixing. In the second set experiments, the detection electrodes were coated with the antibody for *Salmonella* serotype B. Then only *E*.*coli O157* at different concentrations were introduced into the sensors without mixing. The result from these experiments demonstrated that the sensor with *E*.*coli* antigens had a weak signal response while the other sensor with *Salmonella* serotype B antigens has a strong signal response. This demonstrates the device ability to selectively detect Salmonella serotype B.

### Live and dead *Salmonella* cells differentiation

**Fig 12:**
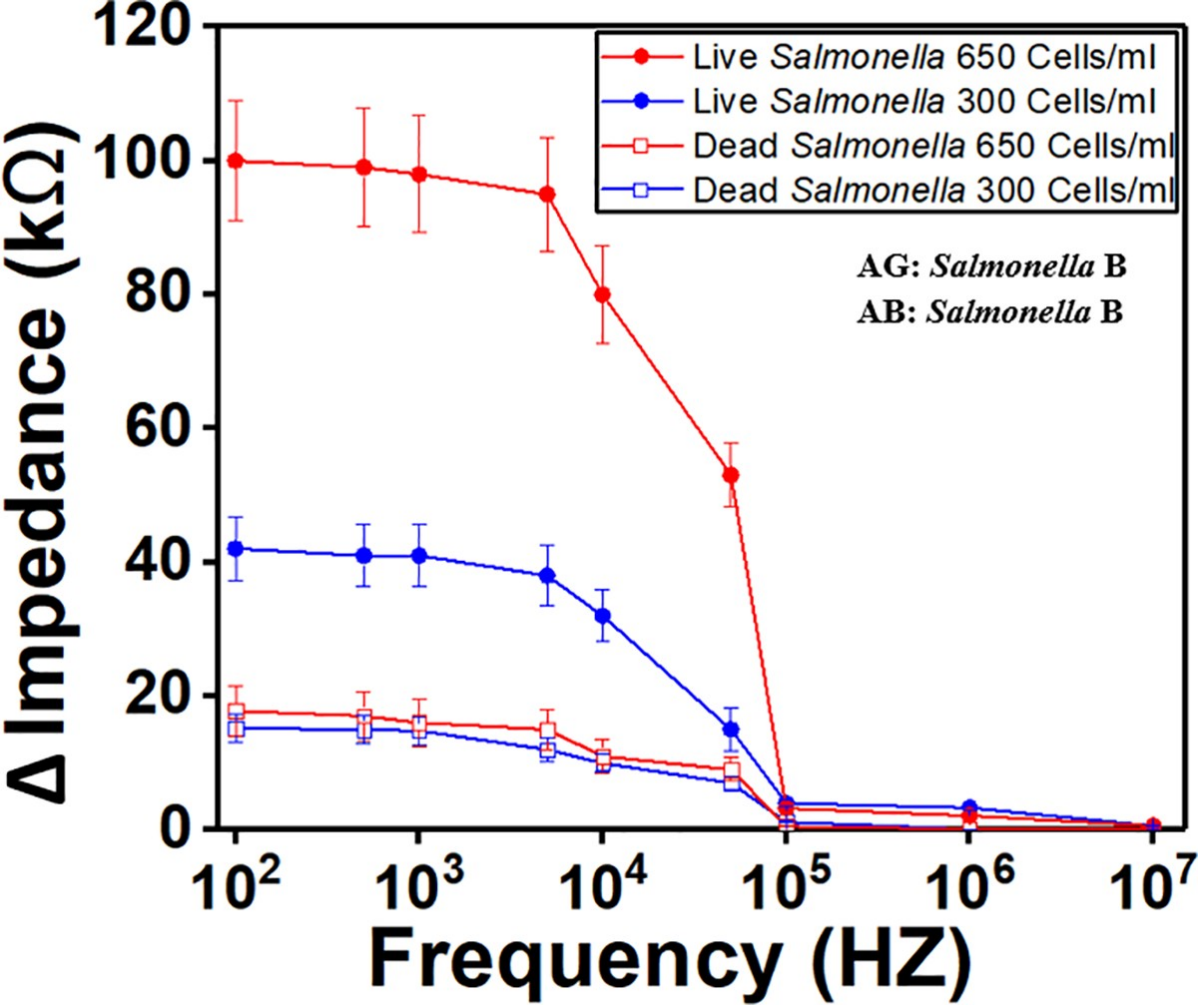
Comparison of live and dead *Salmonella* serotype B for two concentrations. (a) *Salmonella* concentration of 300 Cells/ml and (b) 650 Cells/ml. The results show the sensor’s capability to differentiate between live and dead *Salmonella*. RTE turkey spiked sample and crosslinker were used for these experiments. AB stands for antibody. AG stands for antigen.

The sensor was tested with live and dead *Salmonella* cells to demonstrate its ability to differentiate between them. The *Salmonella* cells were killed by brief exposure to high temperature which resembles real-life event. In each experiment, two sensing regions from single sensor were coated with the same anti-*Salmonella* serotype B, while dead and live bacteria were pumped into the sensor through the antigen inlet. Fig 12 indicates that the signal response for the dead bacteria was very low, which was expected because the membrane of *Salmonella* antigen was damaged and thus lost the function to bind with *Salmonella* antibody.

**Fig 13:**
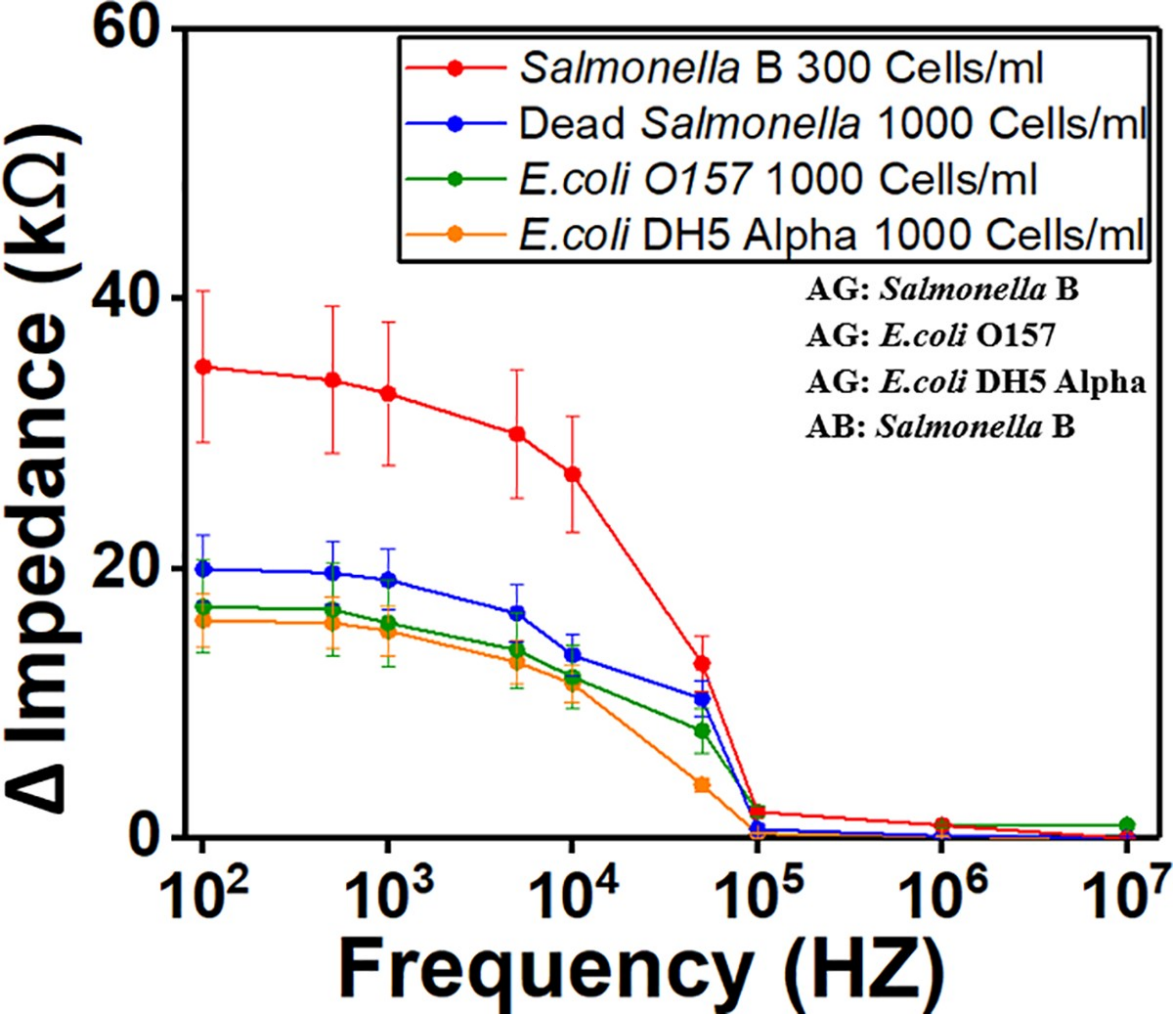
Comparison between low concentration of live *Salmonella*, high concentration of dead *Salmonella* and high concentration of two types of *E*.*coli*. The detection limit of *Salmonella* (300 cells/ml) was plotted with dead *Salmonella* cells (1000 cells/ml), non-specific binding of *E*. *coli O157* (1000 cells/ml) and *E*.*coli DH5 Alpha* (1000 cells/ml). All the samples were spiked in ready-to-eat turkey. Crosslinker was used. AB stands for antibody. AG stands for antigen.

We have plotted the lowest concentration of *Salmonella* (300 cells/ml) in ready-to-eat turkey samples, dead *Salmonella* cells (1000 cells/ml), non-specific binding *E*. *coli* O157 (1000 cells/ml) and *E*.*coli DH5 Alpha* (1000 cells/ml) in Fig 13 to show clearly the difference in impedance values. The figure demonstrates that the lowest concentration of *Salmonella* can be differentiated from high concentration of dead cells and non-specific binding *E*. *coli* O157 and *E*. *coli DH5 Alpha*.

### Testing time

Prior to testing, *Salmonella* antibodies was mixed separately with the cross-linker (Sulfo-LC-SPDP) and was injected into the device via the antibody inlet for 15 minutes. The flow was stopped for 1.5 hours to allow the antibodies-crosslinker to get firmly immobilized on the gold IDE arrays to achieve a good and specific binding between the antibodies and the gold IDE arrays. The channel was then washed for 30 minutes with DI water to remove the unbounded antibodies and other materials, and the impedance of each IDE array was measured. Next, the bacterial testing sample was introduced into the biosensor by syringe pump via the sample inlet. The flow was stopped for 30 minutes to allow the binding between the *Salmonella* antigens and *Salmonella* antibodies. After cleaning the channel with water for 30 minutes, the impedance was measured again, where the impedance change indicated the presence/absence of bacterial cells. We have chosen 30 minutes washing step to ensure that only antibody-antigens are present on the detection electrodes. The other 30 minutes for the antigens to bind to antibody, is not optimum time. We have chosen it in order to maximize the number of antibody-antigens binding. We are currently experimenting to determine the minimum time needed to wait before measuring the antibody-antigens impedance. We expect the detection time to drop much lower. Therefore, the overall testing time is 3 hours and 15 minutes which include the antibody coating time, antigens binding time and the multiple washing step time. It is noted that the biosensor will be used as a disposable device to eliminate the possibility of contamination, per the recommendation of food processing companies including major poultry industry such as Tyson, Cargill, and Pilgrim’s. The device must be pre-coated with antibody cross linker mixture prior to sale. Therefore, the testing time that will be counted is the testing and washing step time, which is 1 hour.

### Electrical Equivalent Circuit and simulation

**Fig 14:**
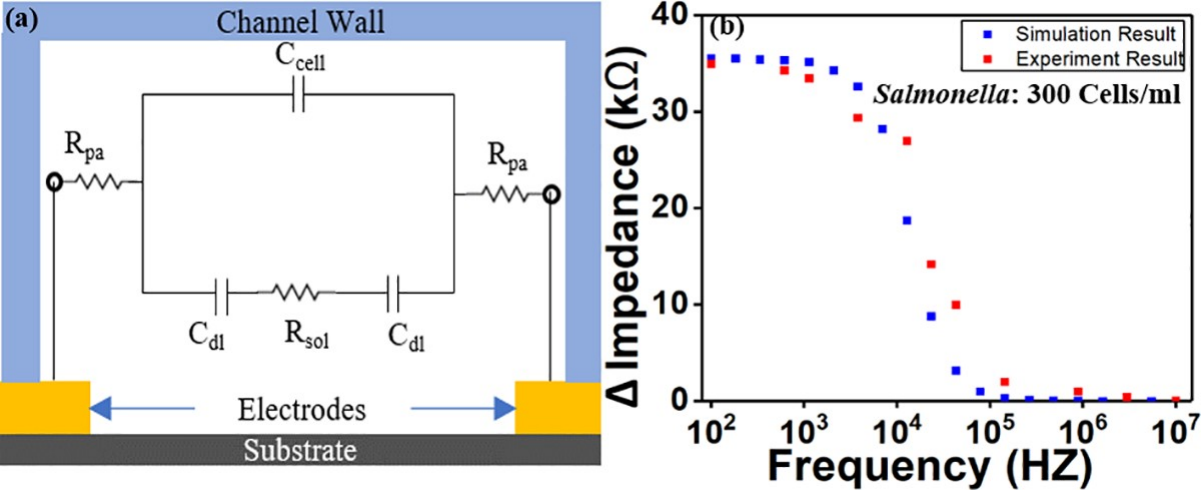
Equivalent circuit and simulation result. (a) Equivalent circuit of the impedance-based biosensor showing the circuit components. (b) The simulation of the equivalent circuit and the experimental results showing a good match of the experimental results with the simulated results for live *Salmonella* of 300 Cells/ml.

An equivalent circuit of the sensor was created to study the impedance response and electrical properties. Fig 14(A). represents the equivalent circuit of the impedance biosensor which consists of two double layer capacitances (C_dl_) connected in series with the solution resistance (R_sol_) and parallel to another capacitance (C_cell_). The electrode pair with each one has an area of A and spacing of G, were placed in parallel. When an AC voltage (*v*) was applied to the electrode pair, an electrical current flow (i) and a solution resistance R_Sol_ would be generated as:

$$R_{\mathrm{Sol}}=\frac{v}{i}\rho_{\mathrm{Sol}}\frac{G}{A}$$ (1)

R_Sol_ represents resistance of the testing solution between the electrodes where ρ_Sol_ is the solution resistivity. R_Sol_ is proportional to the concentration of the pathogen in the tested sample. A thin layer of charged particles was formed on the surface of the electrodes, which would generate capacitance C_dl_. In the equivalent circuit, C_dl_ and R_Sol_ are connected in parallel with the direct capacitive coupling between the electrodes (C_cell_) which depends on the solution dielectric property and the geometry of the electrodes [45]. The parasitic resistors (R_pa_) represents resistance from electrical connections and wires of the circuit which is small and thus can be neglected [45, 46]. R_Sol_ and the two capacitors C_dl_ impedance together will determine the overall impedance, which is expressed as:

$$Z\approx \frac{2+J\omega C_{dl}R_{Sol}}{J\omega C_{dl}}$$ (2)

$$C_{dl}\approx \frac{\varepsilon_{r}\varepsilon_{o}A}{G}$$ (3)

EIS spectrum analyzer software was used to simulate the response of the equivalent circuit. Fig 14(B) shows the results from experiment and simulation (Bode plot) for the electrical equivalent circuit. The simulation value of C_dl_ was calculated to be around 20 nF and R_Sol_ was calculated to be from 30 kΩ to 120 kΩ depending on the concentration of the bacteria. At low frequencies region (below 100 Hz) the impedance response is dominated by capacitive impedance C_dl_. At medium frequency region (100 Hz − 50 kHz) the response is due to both resistive and capacitive components while R_Sol_ is the dominant in this region [46, 47]. At high frequency (above 50 kHz), the impedance becomes pure resistive value [47]. Therefore, the impedance response at high frequencies merely depends on the resistive component of the solution, which means the effect from bacterial cells is insignificant.

The USDA/FDA set a zero-tolerance requirement for RTE poultry products. The testing method must meet the AOAC standard for certification, i.e., 1 cell/325 gr of product. Therefore, a short enrichment step is still required. However, due to the detection limit of our biosensor 300 cell/ml, the total detection time that includes an enrichment will still be less than 24 hours, which is faster than traditional food screen methods like ELISA and PCR. In experiment, by applying the USDA's Laboratory Guidebook which suggests that the enrichment culture is done by inoculating 325 gr RTE poultry into 975 ml BPW at 1:4 dilution. For example, when 10 *Salmonella* cells inoculated on 325 gr RTE Turkey sample, it took approximately 8 hours to reach 400 cells/ml, which can be detected with our biosensing device. For raw poultry products, rather than creating a zero-tolerance standard the USDA maintains a minimum number of samples that must be collected from broiler carcasses [48]. The initial level of bacteria in raw poultry is high, but it is lowered through the processing line by immersing the carcasses in generally recognized as safe (GRAS) substances such as peroxyacetic acid at multiple sites (e.g., Pre-Chill, Chill 1, Chill 2, and Chill 3). At the end of the processing line the remaining bacterial cells must meet the minimum requirement. For example, in a turkey plant, a maximum of 4 samples may test positive for *Salmonella*, in a row of 52 samples [49]. Therefore, the device can be used in the poultry slaughtering plants in the process line at multiple locations to obtain the results in < 1 hr, without the need for a sample enrichment step.

The sensor will be used to determine if the tested poultry sample is contaminated with *Salmonella*, i.e., the device will tell us if the sample is positive or negative to *Salmonella* contamination. It cannot be used to quantify the number of pathogens in the sample. Once the sample is confirmed positive, then other standard techniques can be used to confirm the results and quantify the bacterial cell number.

## Conclusion

This paper has presented design and fabrication of an impedance-based MEMS biosensor for simultaneous detection of two types of *Salmonella* serogroup (type B, and D) detection. The biosensor enables rapid and quantitative detection of *Salmonella* in food source. The device consists of two microfluidic channels with each has an IDE array for bacterial cells detection. This design makes the sensor suitable for simultaneous detections of two different bacterial cells, independently without causing any cross contamination. The results demonstrate that the impedance difference increases when the concentration of target bacteria increases, with the limit of detection found to be 300 Cells/ml. The lowest measured concentration of *Salmonella* can be differentiated from high concentration of dead *Salmonella* cells, and high concentration of non-specific binding *E*. *coli* O157 and *E*. *coli DH5 Alpha*. In addition, the device can selectively detect bacterial cells based on the matching antibody. A detail study of antibody immobilization conditions that includes antibody coating time, antibody concentration, and use of cross-linker has been presented. It is shown that the sensitivity has been improved by 45–60% with the use of Sulfo-LC-SPDP as cross-linker. The binding of *Salmonella* antibody to *Salmonella* antigen is not a factor of antibody concentration after bonding sites on electrode are saturated. 1.5 hours was found to be the optimal antibody coating time. The sensor has also shown its specificity among different *Salmonella* serotypes, selectivity on different types of bacterial cells, and capability to distinguish between dead and live cells with the total detection time of 1 hour.

## Supporting information

S1 Table Impedance reading value for water, antibody, *Salmonella* (300, 500 and 1000 Cells/ml) and *E*.*coli* (1000 Cells/ml). (XLSX)
